# CHAMPION study protocol: children whose mothers are involved in the criminal justice system in Dorset & Hampshire, UK – developing health and social care outcome indicators

**DOI:** 10.1136/bmjopen-2025-100077

**Published:** 2026-06-30

**Authors:** Donna Arrondelle, Naomi Gadian, Lucy Wainwright, Sara Morgan, Natalie Austin, Kathleen Kendall, Julie Parkes, Paula Harriott, Emma Plugge

**Affiliations:** 1 Centre for Population Health Sciences. Department of Primary Care, Population Sciences and Medical Education (PPM), University of Southampton, Southampton, UK; 2 Public Health, Wiltshire County Council, Trowbridge, UK; 3 EP:IC Ltd, Ashford, UK; 4 Unlock Charity, Maidstone, UK

**Keywords:** PUBLIC HEALTH, Child, Clinical Relevance, Health Equity, MENTAL HEALTH

## Abstract

**Abstract:**

**Introduction:**

Each year, approximately 17 000 children will have a mother who is imprisoned in England and Wales. The impact of maternal vs paternal imprisonment is very different; the negative health consequences on children are likely to be much greater if a mother is imprisoned. The number of children affected by non-custodial justice involvement is likely to be far greater given the higher number of women under probation supervision. However, no official statistics or estimates are collected for this population. The purpose of the CHAMPION (children whose mothers are involved in the criminal justice system in Dorset & Hampshire, UK – developing health and social care outcome indicators) study is to develop a better understanding of the evidence of the impact of maternal contact with the CJS (Criminal Justice System) on children and to establish a set of core outcome indicators for monitoring and evaluating affected children’s health and well-being.

**Methods and analysis:**

(i) A scoping review of the health impacts of maternal vs paternal imprisonment, (ii) qualitative focus groups and interviews with adults whose mother was imprisoned when they were children and children of mothers who have either been or currently are in prison or have received community order sentences. Qualitative interviews with a range of professionals working with these children, (iii) an outcomes workshop with justice-involved mothers and professionals working with the children and families of justice-involved mothers, and adult children who experienced maternal imprisonment.

**Ethics and dissemination:**

Ethical approval from the University of Southampton’s Faculty of Medicine Review Panel (Ref: 76946) and His Majesty’s Prisons and Probation Service (HMPPS) National Research Committee (Ref: 2023-135). Publishing in peer-reviewed journals, presenting at research conferences, round table events and community-based workshops. The study’s peer researchers and lived experience leaders will enable the dissemination of findings beyond academic audiences and policy makers to third sector CJS organisations and individuals with lived experience.

**Strengths and limitations of this study**

- It provides a synthesis of the evidence on an underserved population currently underexplored in health research, of children of maternal imprisonment.
- It advances a child-centred approach to develop health and well-being outcomes for children of maternal imprisonment for health monitoring and evaluation purposes.
- Lived experience/patient and public involvement and engagement is embedded in every aspect of the study from the co-design through to dissemination of this study.
- The outcomes to be developed by this study are relevant to child patients, parents of child patients, clinicians and non-clinical staff working in health and social care settings. The scoping review included published studies but excluded grey literature.
- A potential limitation of this study is the range of extant literature which is largely USA based and of limited quality.

## Introduction

 The imprisonment of a parent is an adverse childhood experience (ACE), known to have a strong association with poorer long-term health and well-being.^1^ Each year, in England and Wales, an official estimated 191 912 children are affected by parental imprisonment.^2^ This number is an estimate because there is no systematic collection of data on these children. Of these children, about 17 000 will have a mother who is imprisoned.^3^ Although numerically smaller, the children of imprisoned mothers are more likely to suffer adverse impacts than those of imprisoned fathers.^4^ Central to this suggested differential impact is the fact that it is likely that the mother, not the father, is likely to have been the child’s primary carer prior to her imprisonment^5^ and so the subsequent disruption to the child’s life that follows her imprisonment is likely to be profound. In 95% of cases, when a father is imprisoned, his children remain with their mother, usually in the family home. It is very different when a mother is imprisoned: most children will not be cared for by their father but will be removed from the family home either into statutory care (the majority) or to be looked after by grandparents (about 40%).^3^ Thus, these children are likely to lose both their mother and their home. This physical displacement and the loss of a familiar place is compounded by the fact that they are likely to have moved area and so have to start a new school, develop new friendship networks and so forth. They may even be separated from siblings.^6^ Furthermore, for many women, being imprisoned results in the loss of their accommodation and so for the children, the loss of home is often permanent. This lack of stable accommodation often compounds imprisoned mothers’ struggle to regain custody of dependent children on release.^7^ Visits to see an imprisoned parent can entail travelling long distances further complicating contact possibilities.^8^ These findings are largely based on small studies and therefore, it is unclear to what extent generalisable conclusions may be drawn with children experiencing parental imprisonment beyond the specific groups observed.

Regardless of which parent is imprisoned, separation through imprisonment can generate significant family disruption, uncertainty and instability for the entire family, not only the children as well as the carers.^3^ The grief that children experience being unable to see or speak to their imprisoned parent can be profound.^9^,^11^ It is not surprising that the available evidence suggests that parental imprisonment has a negative impact on the mental and physical health of these children.^3^ However, the evidence is not of high quality and most of it comes from the USA. As mentioned, to date, the research is largely qualitative with small, non-representative samples. In addition, the quantitative studies are usually cross-sectional surveys although a few studies look at longitudinal data.^4^ Furthermore, most of the studies focus on the impact of the imprisonment of the father, not the mother, probably because women make up a small proportion of imprisoned people, about 6% globally.^12^ Nonetheless, the studies suggest wide-ranging impacts on the health of children whose parents are imprisoned, including grief and depression, post-traumatic stress disorder,^13^ behavioural problems and substance use.^14^ Chronic stressors related to maternal imprisonment have been found to be especially acute when accompanied by paternal imprisonment.^15^ However, the authors of one study suggested that the association between imprisonment of a parent and adverse outcomes in adolescence can be accounted for by well-established social and familial risk factors such as socioeconomic status and parenting style, and that imprisonment may not in itself increase the risk for child behaviour and substance use problems.^16^ This study, however, only looked at paternal imprisonment and the impact on children of imprisoned mothers is, as already outlined, likely to be different and more severe. Other researchers suggest that it is the environment that children are living in prior to parental imprisonment which increases the likelihood of poorer health outcomes.^17^

Various outcome measures have been examined in a handful of studies relating to parental imprisonment and education attainment, children’s imprisonment, language and employment and health and well-being. The evidence on the health impacts for maternal and paternal imprisonment is mixed.^18^ Poehlmann-Tynan and Turney’s synthesis of longitudinal studies examining health implications of children looked at the age that a child is faced with parental imprisonment. The study found that parental incarceration had a negative impact on mental health across all age groups but the effects appeared stronger for those in early adulthood than those in early and middle childhood and adolescence. However, the authors highlighted inconsistency across the research findings. They also highlight the need for a greater understanding of the effects relating to frequency of justice involvement of the parent(s) and the age-related effects.^19^

A systematic review and meta-analysis examining association between post-traumatic stress disorder (PTSD) in children with experience of parental imprisonment found a higher prevalence of diagnosed PTSD in these children compared with children without experience of parental imprisonment; the prevalence was even higher for those who had experience of maternal imprisonment compared with those with paternal imprisonment experience.^13^ Two other studies focusing on depression and suicide found significant links between maternal imprisonment and higher levels of depression and suicide in the affected children.^20^ However, Geller *et al* found no significant difference for children with maternal imprisonment and those without any imprisoned parent.^21^

A variety of study methods and designs including the different measures used to understand health and well-being outcomes make comparison of findings across studies difficult. For instance, a study by Le *et al* reviewing the early onset of sexual intercourse for children with parental imprisonment used a threshold age of 12 years for measurement while a study by Nebbit *et al* cited 13 years as the threshold age.^22 23^ Notably, many studies examine parental imprisonment without differentiating between the imprisonment of mother or father. This is an important omission given that maternal imprisonment is likely more disruptive to a child’s life as mothers are more often the primary caregiver of the child prior to incarceration than the father.

The UK Government’s Female Offender Strategy acknowledges the ‘disproportionate impact on children and families’ of maternal as opposed to paternal imprisonment.^24^ However, while acknowledging this, the strategy is concerned with the evidence that children of imprisoned mothers are much more likely to go on to pursue criminalised conduct in the future^25^ and does not examine closely the health and well-being impacts of maternal imprisonment on these vulnerable children. Similarly, Lord Farmer’s 2019 Review, ‘The Importance of Strengthening Female Offenders’ Family and Other Relationships to Prevent Reoffending and Reduce Intergenerational Crime’, focuses on the impact on the imprisoned women of separation from their children rather than the impact on the children themselves.^26^ Shona Minson, a leading criminologist in this area, is a vocal advocate for the need to reduce the number of women with children going to prison because of the ‘corrosive’ impact on their children.^27^ The Female Offender strategy endorses training material she has developed, ‘Safeguarding Children When Sentencing Mothers’, which is aimed at magistrates and judges who sentence women in the courts and raises awareness of the diverse implications of maternal imprisonment for children. However, it falls short of endorsing Minson’s recommendation that magistrates who wish to impose a custodial sentence on a woman with dependent children should refer the decision to a Crown Court judge^28^ and therefore, many thousands of women each year continue to be sent to prison for non-violent crimes for sentences of a few weeks only,^29^ with likely harmful consequences on many thousands of children each year. The recent UK government’s consideration and implementation of some of the Independent Sentencing Review recommendations are under way, including efforts to use community sentences instead of custody for sentences under 12 months. The impact is yet to be seen.

It is against this backdrop that Hope Street, a residential community-based alternative for women facing imprisonment and for women encountering the legal system, has emerged. Unlike prison, where children are separated from their mothers when custodial sentences are given, children are able to stay with their mothers at Hope Street, where assessed appropriate. Hope Street also offers support to re-establish contact with children where they have been separated from their mothers prior to Hope Street. Opened in June 2023, in Southampton, UK, it is the first of its kind in England and intended to be the start of a nationwide residential network of trauma-informed designed and delivered provision for justice-involved women and their children. The idea that children and mothers are enabled to stay together is key to Hope Street. One Small Thing, the charity funding Hope Street, states that it is ‘designed with women and children at its heart’. It aims to ‘Reduce the number of children being taken into care as a result of their mother receiving a custodial sentence’ and there will be provision for women to live with their children, ‘subject to an assessment of suitability on a case-by-case basis’.^30^

This study addresses two gaps in the literature relating to this invisibilised population, (i) a limited knowledge base on the differential impacts between maternal imprisonment and paternal imprisonment, and (ii) a lack of understanding of the health and well-being needs and priorities of children with maternal imprisonment experience. These gaps are addressed first, through a scoping review, synthesising the extant evidence differential impacts between maternal imprisonment and paternal imprisonment and second, through qualitative work leading to the development of child-centred outcome indicators. The purpose is to develop a set of child-centred indicators for use by practitioners and clinicians working with children with maternal imprisonment experience.

### Methods and analysis

The study objectives are:

1. To establish the current evidence on the differential health impacts on children of maternal and paternal imprisonment.
2. To understand how maternal imprisonment affects the health and well-being of children and what aspects of well-being are of importance to the children themselves.
3. To explore with the affected children and relevant professionals, potential service interventions to minimise and mitigate harms.
4. To establish a set of children’s health and well-being core outcomes for monitoring and evaluating the impact of specific criminal justice interventions applied to their mothers.

### Study design

This mixed methods study combines a desk-based scoping review (addressing objectives 1 and 2) and field site qualitative data gathering with desk-based qualitative analysis (addressing objectives 2, 3 and 4) in a staged approach building from the scoping review to outcome indicators formulation and agreement.

### Project status

The study commenced in April 2023 and will conclude in March 2026. At the time of writing, objective 1 has been met, 2 and 3 are under way and 4 is yet to commence.

### Strand 1: Scoping review

A scoping review of the health impacts of maternal vs paternal imprisonment was conducted with the aim to enable a better understanding of the differential impacts on affected children and to secure clarity on what outcomes have been measured and where the evidence gaps remain. It will inform both the qualitative work in Strand 2 and outcomes indicator development workshop in Strand 3. The thematic findings will shape both the focus group and interview schedule design as well as the development of outcome indicators.

### Conduct

We followed the five stages of the Arksey and O’Malley methodology for scoping reviews:

1. Identifying the research question.
2. Identifying relevant studies.
3. Study selection.
4. Charting the data.
5. Collating, summarising and content analysis of documents included.

The research question for this scoping review was, ‘How do the physical, mental, behavioural and well-being health, along with healthcare service use differ between children who experience their mother being imprisoned, compared to those who experience their father being imprisoned?’ The question was generated in response to the disparate knowledge on physical, mental, behavioural and well-being health, along with healthcare service use for children with parental experience of imprisonment and knowledge gap surrounding differential impacts between incarcerated parent type. Until now, there has been no systematic synthesis of the existing evidence of outcomes relating to children experiencing maternal vs paternal imprisonment in studies of the same population. The term ‘prison’ represents detention facilities housing both on remand and convicted people and includes prisons, police holding cells, pre-trial detention, closed youth institutions, and camps where drug users are forced into mandatory labour as means of rehabilitation. ‘Imprisonment’ relates to the placement of an individual in prison.

We searched the following electronic databases: Medline, Embase, CINAHL (Cumulative Index to Nursing and Allied Health Literature, Cochrane), PyschINFO, Web of Science, Delphis and IBBS (International Bibliography of the Social Sciences). There were no limits on date, language or study design/type of publication. The search terms were broad to maximise sensitivity. These included: imprison* or incarcerat* or jail* or prison* or gaol*; Child* or ‘young person’ or adolescen* or teen* or youth* ; Maternal* or mother* or mum* or mom* or mam* ; paternal* or father* or dad* or papa*. Review articles were not included, but their reference lists were hand-searched. We also searched the reference list of included articles. To ensure robust mapping of measurable population-level outcomes and associations, qualitative studies were excluded from scoping. The inclusion criteria of prevalence studies, cross-sectional studies, cohort studies, case control studies, and surveys were most appropriate to enable a comparable epidemiological evidence base. Grey literature was excluded given the aim to review the published evidence about the health impacts of maternal vs paternal imprisonment to enable a better understanding of the differential impacts on affected children and to identify where gaps in the evidence remain.

Two members of the evaluation team screened the title and abstract of each record to determine inclusion status. Full texts of all records warranting assessment of inclusion by the team were obtained. A second screening of the full text of each record was conducted and records were excluded at this stage if found not to meet the eligibility criteria.

Table 1 below outlines the inclusion and exclusion criteria.

**Table 1:** Study inclusion and exclusion criteria

Inclusion	Exclusion
Participants	
Any children or adolescence who experienced their mother and father being imprisoned	If parent was detained in immigration removal centres or secure psychiatric units or was a prisoner of war
Male or female	
Intervention	
Experience of maternal imprisonment or father imprisonment, or both, within the same study population. The comparison group may be experiencing no parental imprisonment	Only focused on maternal or only paternal imprisonment
Outcome	
Any health condition including: Physical health Mental health Behavioural health, for example, behavioural challenges and sleeping. The paper must report health outcomes for children who have experienced maternal imprisonment and children who have experienced paternal imprisonment	Not a health condition, such as income, homeless, school achievement educational level sporting achievement involvement in the criminal justice system family relations
Study	
Prevalence studies, cross-sectional studies, cohort studies, case control studies, surveys	Policy, opinion, qualitative studies, review articles. (Review articles were excluded but their reference lists were used to check for any missed papers)
Any country, any date of publication, any language	
Published and un-published data	

### Analysis

We charted, summarised and analysed the content of all the papers included. We created a charting spreadsheet and used it to collect and sort key pieces of information from each record. We conducted a trial charting exercise of five records as recommended by Levac and colleagues.^31^ Disagreements around allocation of content were resolved through team discussion. We also appraised the quality of the included papers using the National Institute of Health (NIH) Assessment tool^32^ (*).

Data were charted in a spreadsheet under the headings: author, month and year, title, population, key findings. NG, AD and EP reviewed a selection of full texts. The data were collated and organised thematically into shared categories, including mental health, sexual health, sleep and diet. NG, AD and EP independently applied the NIH Assessment tool to every included study. 25% of the papers were discussed assessing their quality and where there were differences or uncertainty, consensus was reached through discussion.

The scoping review’s thematic findings will be used to inform the outcomes workshop (Strand 3) and written up for scientific publication.

### Strand 2: Qualitative work

#### Focus groups

The aim of this work is to begin to understand the experiences of adult children in relation to their childhood experience of maternal imprisonment. This data will then inform the development of a child-centred interview schedule. The emergent themes from these focus groups will be incorporated into the interview schedule design to be conducted with children with experience of maternal separation through maternal justice involvement with particular focus on health and well-being needs.

#### Focus group participants

We will conduct 2–4 focus groups with a total of 12–24 adults whose mothers were imprisoned when they were children. We will use a lived experience expert panel (LExP) with individuals whose parent was imprisoned when they were a child to identify possible participants. The panel is already established, previously working across different studies with various members of the study team. These women are members of a now disbanded network, the Prison Reform Trust’s Prisoner Policy Network (PPN) formerly headed by one of the research team, Paula Harriott. The network’s membership was comprised of people who were currently in, or used to be in prison, their families and organisations that support them with over 800 prisoners involved. PPN aimed to share the views of people with experience of living in prison with those involved in prison policy development nationally through research, consultation and reports. We will use purposive sampling to ensure people from a range of ages, ethnicities, health and family circumstances are included. We will aim for small groups of no more than 6 participants to enhance a sense of psychological safety through a small group setting.

#### Eligibility

Adults over the age of 18 years who have both experienced and are able to recall the imprisonment of their mother at some point during the course of their childhood or adolescence are eligible to be included. Individuals will be excluded if they are unable to give informed, voluntary consent for participation in the study. In accordance with the Mental Health Capacity Act, individuals deemed unable to provide informed consent due to current mental health issues and anyone who was in acute crisis, whether this was related to addiction, their own mental health or any other factor in their life. This judgement will be made by the researchers conducting the focus groups.

#### Setting

These focus groups will be conducted online (MS Teams).

#### Conduct

There will be a facilitator and co-facilitator from the research team. Using a semi-structured qualitative approach, we will explore their experiences of maternal imprisonment and their perceptions of its impact on their health and the reasons for this. We will also seek an understanding of what helped their health and well-being, specifically probing about the role and effectiveness of health and social care services. We will ask them to consider what they believe to be the important aspects of health and well-being for children that should be measured.

#### Analysis

The focus groups will be recorded and transcribed. We will use thematic analysis.^33^ We will read and re-read the data before coding and classifying. We will use NVivo software to facilitate data coding and organisation into conceptual categories, ‘themes’. Anticipated and emergent themes in the data will be analysed using constant comparison and examination of deviant cases. The findings from these focus groups will be used to inform the development of an interview schedule for in-depth interviews with children and professionals.

#### Interviews

The aim of this work is to inform the development of outcome indicators through gathering children-centred data relating to experience of maternal separation through maternal justice involvement with particular focus on health and well-being needs. The interview schedule is to be developed based on the emergent themes from the previous focus groups.

##### Interview participants

We will conduct in-depth interviews with children aged over 7 years who are (i) currently resident in Hope Street or whose mother is resident in Hope Street and they visit her there and children of former resident women, and (ii) children with experience of maternal separation through maternal justice involvement, nationally. With the children of women living at Hope Street, their mothers have faced criminal justice sentencing, some being imprisoned and others being served suspended/community order sentences. While the children may not have experienced their mother being imprisoned, they will have experienced their mother’s arrest and subsequent court proceedings and other processes of community punishment, such as probation supervision and other court order requirements. We will explore the children’s experiences of their mother living at Hope Street and their perceptions of its impact on their own health and the reasons for this. We aim to recruit approximately 10 child interviewees.

We will also interview (iii) a range of professionals such as support workers and child care workers at Hope Street, external health staff, social workers and teachers of school age children resident at Hope Street. We aim to recruit approximately 12 participants. We will explore their perceptions of Hope Street and its impact on the well-being of the children living there.

##### Identification of participants

Children residing at Hope Street and elsewhere will be identified through discussions with mothers resident at Hope Street, and with mothers formerly resident at Hope Street as well as through professional contacts with NGOs working with children and families experiencing justice involvement, nationally. We will take a purposive sampling approach to ensure that a breadth of participant ages, experience and ethnicities.

##### Assessing capacity and obtaining informed consent

Initial conversations will be held with the mother of the child to gain their perspective on the child’s capacity for interview participation. If positive, the researcher will sit with the child in the mother’s presence and talk through the age-appropriate information and assent form designed for Key Stage 2 level (ages 7–11) and information and consent form designed for Key Stage 3 level (ages 11–14).

### Setting

These interviews (i, ii and iii) will be conducted online (MS Teams), in person at Hope Street or at the professional’s place of work and where children have been recruited through NGO organisations in person in a private room at NGO premises.

### Conduct

The interviews will be recorded and transcribed. We will use thematic analysis.^33^ We will read and re-read the data before coding and classifying. We will use NVivo software to facilitate data coding and organisation into conceptual categories, ‘themes’. Anticipated and emergent themes in the data will be analysed using constant comparison and examination of deviant cases.

The thematic findings will be used to inform the outcomes workshop (Strand 3). We will share these with the stakeholder group participants invited to the outcome indicators workshop in a written report ahead of the workshop to consider the development of indicators and to be discussed at the workshop.

### Strand 3: Workshop on outcome indicators

The findings from the scoping review (Strand 1) and all aspects of the qualitative work (Strand 2) will feed into the outcomes development workshop (Strand 3).

We will run a half-day workshop with a range of local stakeholders focusing on health and well-being outcomes to be measured. The aim of this workshop is to agree a set of children’s health and well-being core outcomes for monitoring and evaluating the impact of specific criminal justice interventions applied to their mothers.

### Participants

We will invite relevant professionals such as GPs (General Practioners), paediatricians, child psychiatrists, health visitors, school nurses, social workers and teachers. We will also invite staff from Hope Street and people with lived experience of their mother being imprisoned when they were a child. We anticipate the number of participants to be approximately 20–30.

### Setting

The workshop will be conducted in person at Hope Street in a private room.

### Conduct

Following a modified nominal group technique, we will present the findings of Strands 1 and 2 to all participants before using small groups to stimulate discussion about important outcomes relating to physical, mental and social well-being (including educational experiences and progress) and to reach consensus on a list of prioritised outcomes. A modified nominal technique will be used to reach consensus through a structured and multistep process to generate themes, discuss and rank them within a facilitated group meeting setting.^34^

### Analysis

After the workshop, we will analyse the data obtained and draw up a list of outcomes. This will be circulated by email to participants for final agreement and refinement if necessary.

### Patient and public involvement statement

This is unique work not simply because this has not been done before but because of the level of patient and public involvement and engagement (PPIE). People with lived experience of imprisonment and people with lived experience of having an imprisoned mother as a child are members of the core research team, informing study design and developing study materials from its inception, conducting the research, and are co-authors on this paper. The study has an LExP with upwards of 10 members to substantively input on research design, all study materials, analysis and dissemination (led by LW and PH). These women are part of an existing network, previously established as the Prison Reform Trust’s Prisoner Policy Network PPN (Prisoner Policy Network) formerly headed by one of the research team (PH). Women resident at Hope Street will also be invited to become members of the LExP (Lived Experience Panel). The LExP will also provide valuable guidance on how best to engage with the children and their mothers on the project. The membership are financially reimbursed for their contribution in line with NIHR (National Institute of Health Research) PPIE guidance. Support and training for members is provided by lived experience leaders in the core research team. Experts by experience also comprise 30% of the study’s Independent Advisory Group.

#### Ethics and dissemination

The study will be conducted in compliance with the approved protocol and the principles of Good Clinical Practice, good practice research guidance from The Department of Health and Social Care or the Ministry of Justice as relevant. The researchers will comply with the full protocol and applicable national regulations, including the Mental Health Capacity Act (2005). The study will be conducted to protect the human rights and dignity of the participants as reflected in the 2013 version of the Helsinki Declaration.^35^ Ethical approval was obtained from the University of Southampton’s Faculty of Medicine Review Panel (Ref: 76946) and His Majesty’s Prisons and Probation Service (HMPPS) National Research Committee (Ref: 2023-135).

A findings report will be presented to the funder which will be translated into different formats for different stakeholders, including the MoJ, HMPPS and Hampshire and Dorset Constabularies. We will share the findings through the networks of the study’s peer researchers and lived experience leaders targeting the third sector and civil society. We will submit papers to high impact peer-reviewed international journals and present findings at academic and professional conferences. We shall use our links with NHS (National Health Service) England, Department of Health and Social Care, and the Ministry of Justice to inform policy makers and commissioners of the research findings and explore creative approaches to enhance dissemination across all audiences.

## Discussion

This study will be the first to build the knowledge base surrounding children of mothers who have been or currently are in prison to understand what aspects of their health and well-being they consider important and why. As an invisibilised cohort, the health needs of children with experience of maternal imprisonment have historically been underserved by health and social care services and this study works to redress this. It will provide important information to practitioners working with these women and their children supplying a standardised set of child-centred health and well-being outcome measures. These outcome measures will enable practitioners to better understand the specific health and well-being needs of this population.
