# Supplementary material for: CHAMPION study protocol: children whose mothers are involved in the criminal justice system in Dorset & Hampshire, UK – developing health and social care outcome indicators
Source: BMJ Open. 2026 Jun 30;16(6):e100077. doi: 10.1136/bmjopen-2025-100077 (PMC13331118; doi:10.1136/bmjopen-2025-100077)
Supplement: Supplementary file 1 [file bmjopen-2025-100077.reviewer_comments.pdf]

## PEER REVIEW HISTORY

BMJ Open publishes all reviews undertaken for accepted manuscripts. Reviewers are asked to complete a checklist review form and are provided with free text boxes to elaborate on their assessment. These free text comments are reproduced below.

### ARTICLE DETAILS

**Title (Provisional)**

CHAMPION study protocol: children whose mothers are involved in the criminal justice system in Dorset & Hampshire, United Kingdom: developing health and social care outcome indicators

**Authors**

Arrondelle, Donna; Gadian, Naomi; Wainwright, Lucy; Morgan, Sara; Austin, Natalie; Kendall, Kathleen; Parkes, Julie; Harriott, Paula; Plugge, Emma

---

### VERSION 1 - REVIEW

---

| | |
|---|---|
| **Reviewer** | **1** |
| **Name** | **Bradshaw, Daragh** |
| **Affiliation** | **University of Limerick, Psychoogy** |
| **Date** | **04-Mar-2025** |
| **COI** | **None** |

---

Thank you for the opportunity to review the manuscript. It is a very important and under researched area particularly outside of the US context.

That being said there are a number of areas that I think need to be addressed. Overall far more detail is needed in the literature review, theoretical perspective, methods, and proposed analysis.

For example, there needs to be a more thorough engagement with previous research in the introduction [for example page 3, lines 34-41 list a number of outcomes but no supporting references]. The assumption of the protocol is that maternal incarceration is more detrimental than paternal incarceration. Previous evidence suggests this may not be so clear cut. Maternal incarceration research often supports the null hypothesis as the issues surrounding maternal incarceration are so great that the incarceration is the culmination of a series of adverse life experiences that have already disadvantaged the mothers and children involved. This would suggest that in maternal incarceration the incarceration is a consequence rather than a predictor. possibly reflecting this preexisting disadvantage, children are often more likely to be involved in the care system prior to maternal incarceration rather than in the context of paternal incarceration.

You mention that there are few longitudinal papers and non-US studies, it would help perhaps to reference the studies that do exist.

Importantly, you do not offer a mechanism for change. You outline that there is a detrimental outcome of maternal incarceration on the children but do not draw on the evidence or a theoretical approach for why or how this might occur. You end with an outline of an intervention that will address this proposed connection but without a theoretical perspective it is not clear how the intervention alleviates the negative outcome, or why you chose this intervention over other potential alternatives.

Overall the introduction needs a lot more engagement in the literature to prepare us for the subsequent research questions.

Study 1. as indicated above, it is not clear how you developed the research question. The introduction does not go into enough detail to justify it. Additionally, Far more detail is needed on how you are going to conduct the search and analyses in this study.

Also, as many studies will present null findings they may not be captured by published articles alone, why are you excluding grey literature searches?

How will findings be used to inform outcome workshops? what if the findings are that there is no difference between incarcerated parent status, or if there are incomparable studies and so no conclusions can be drawn?

Relatedly, you say you are not going to restrict based on study design, how do you compare groups with different study designs, or are you only searching for studies that compare group already?

Do you have search terms already prepared, if so what are they?

What are the inclusion and exclusion criteria?

What data will be extracted to conduct the group comparison?

How will the analysis be conducted?

There are many other methodological questions will arise as you elaborate on the above questions, more detail on what exactly you intend to do is needed in this study.

On a minor note, what is the CHAMPION report that is being informed by this?

Study 2. you indicate that you will use a panel of individuals who have experienced maternal incarceration to identify individuals who have experienced maternal incarceration. It is a good practice to include individuals with lived experience on the panel but it does not get around the issue of how are you going to identify and recruit members to the panel? why are the panel not included in the interviews?

How are you going to assess homogeneity and heterogeneity within and between groups and what does it add to separate groups in this way? you mention you will split this across gender but a man who has experience maternal incarceration age 7 possibly will have more

in common with a women who also experienced maternal incarceration aged 7 rather than another man who experienced maternal incarceration at 16. How big are these groups?

Again going back to the literature review, what gap in the literature do these focus groups address and what is the specific research question of this study?

Study 3 looks at the experiences of children whose mother is resident in Hope Street. The main area of clarification is what is being addressed here and how does it relate to the previous two studies? The stated aim of Hope street is to reduce the number of children taken into care, what is the aim of this study? is it to demonstrate children are better off or that children are not being taken into care? More work is need to explain how all of these studies are linked together or they need to be submitted as separate protocols with separate aims and objectives.

Study 4, looks at professional perspectives, similar questions are present to study 3 above. Additionally, as you are recruiting professional stakeholders, is this a sort of process evaluation? are you recruiting professionals who have experience of other contexts? what is the over all aim of this arm of the protocol and how does it link back to previous studies?

Study 5. This is an interesting task, why is it being done at the end rather than the beginning? If you identify outcomes to be measured now how are you going to measure them if you have already collected the data?

There are a lot of proposed studies for such a short protocol. You need to engage in the previous literature to establish current knowledge, in a lot of areas as you have 5 different studies here, and then identify how these studies address a specific gap. It would also help the reader if you identified how the studies are linked and inform each other. Also, perhaps more detail on what a Nominal Group Technique is, I am aware of Depphi and World Café but had to look up what was involved in NGT

This is really important research and I commend the ambition here. I look forward to reading about how it progresses, but there is a lot more detail needed in this protocol for a reader to understand what is proposed and why.

---


| | |
|---|---|
| **Reviewer** | **2** |
| **Name** | **Holaday, Louisa** |
| **Affiliation** | **Mount Sinai School of Medicine** |
| **Date** | **27-Mar-2025** |
| **COI** | **None** |


---

This is very important work, and an intriguing study design; however, the protocol leaves gaps in my understanding of how the proposed projects will address the gaps in the literature around children of imprisoned mothers and the overall design is unclear. While the

topic itself does seem important, the introduction does not clearly set up how the study will address gaps in our understanding; it is unclear how the different “strands” of the protocol will be combined; and justification for the planned approach is not adequately provided. Below, I have made recommendations to help strengthen the manuscript, grouped into major and minor revisions.

Major:

- The introduction is compelling regarding the potential harms of having an imprisoned mother, and the large gaps in understanding of children’s needs; however, it does not clearly set up why the study proposal focuses on adult children of imprisoned mothers and current children of mothers at Hope Street – which appears to be an incarceration alternative. Please re-format the introduction to set up clear gaps in the literature that are then directly addressed by what is proposed in this study protocol.

- The scoping review seems to be more of a background literature search to inform the main study; it is not clear to me it should be described here.

- Further, the scoping review protocol is limited – what data will be collected? How will this inform strands 2 and 3

- Please provide justification for the study design, particularly strands 2 and 3. For example, why 2-3 focus groups? How will you decide? What is the goal of the workshop? How many people will be there?

- The descriptions of all strands lack detail, please add.

- What is the goal of the in-depth interviews? What data are you hoping to obtain from 8+ year olds?

- What is the goal of focus groups and work shops?

Minor:

- Abstract re: 17,000 children, please contextualize – is this in the UK broadly or a particular area?

- Intro, first paragraph, is there evidence to support that children are likely to move area and start a new school if their mother is imprisoned? If so, cite.

- The use of “etc.” throughout the document feels inappropriate.

- Dates of the study missing

---

## VERSION 1 - AUTHOR RESPONSE

| **Requested revisions** | **Response to reviewer suggestion** |
|---|---|
| **Reviewer 1 comments** | |

| | |
|---|---|
| Overall far more detail is needed in the literature review, theoretical perspective, methods, and proposed analysis.<br><br>For example, there needs to be a more thorough engagement with previous research in the introduction [for example page 3, lines 34-41 list a number of outcomes but no supporting references]. The assumption of the protocol is that maternal incarceration is more detrimental than paternal incarceration. Previous evidence suggests this may not be so clear cut. Maternal incarceration research often supports the null hypothesis as the issues surrounding maternal incarceration are so great that the incarceration is the culmination of a series of adverse life experiences that have already disadvantaged the mothers and children involved. This would suggest that in maternal incarceration the incarceration is a consequence rather than a predictor. possibly reflecting this preexisting disadvantage, children are often more likely to be involved in the care system prior to maternal incarceration rather than in the context of paternal incarceration.<br><br>You mention that there are few longitudinal papers and non-US studies, it would help perhaps to reference the studies that do exist. | Thank you for your careful consideration. We have developed the literature review along the lines suggested, presenting more studies and emphasising the mixed evidence. This content is in the expanded Introduction section. |
| Importantly, you do not offer a mechanism for change. You outline that there is a detrimental outcome of maternal incarceration on the children but do not draw on the evidence or a theoretical approach for why or how this might occur. You end with an outline of an intervention that will address this proposed connection but without a theoretical perspective it is not clear how the intervention alleviates the negative outcome, or why you chose this intervention over other potential alternatives.<br>Overall the introduction needs a lot more engagement in the literature to prepare us for the subsequent research questions | The proposed study is a method to develop quality indicators rather than an intervention (i.e. application of screening tool) to alleviate the negative outcomes. In theory of change terms, this study offers a potential input to feed into future interventions, contributing to a mechanism of change through better identification of health challenges using the outcome indicators developed in this study. |
| Study 1. as indicated above, it is not clear how you developed the research question. The introduction does not go into enough detail to justify it. Additionally, Far | This is a single study with different components rather than five studies. In Strand 1 (not Study 1 as this is a single study), we have developed the introduction and we |

| | |
|---|---|
| more detail is needed on how you are going to conduct the search and analyses in this study. | hope it is now clear how we have arrived at the research question and why the study is needed. |
| Also, as many studies will present null findings they may not be captured by published articles alone, why are you excluding grey literature searches? | We focused on published studies as this was achievable within the available resources. It should be noted that a large proportion of the included studies did indeed present null findings. This is an acknowledged limitation of the study as stated in the strengths and limitations section. |
| How will findings be used to inform outcome workshops? what if the findings are that there is no difference between incarcerated parent status, or if there are incomparable studies and so no conclusions can be drawn? | The findings from the scoping review will be used to provide the status of the evidence to the stakeholders ahead of the development outcomes workshop. The unclear conclusions support the necessity for better understanding of the population's needs i.e. the purpose of the indicator development workshops. The scoping review summarises the various measures that studies to date have used. This basic information is necessary for the outcomes workshop. |
| Relatedly, you say you are not going to restrict based on study design, how do you compare groups with different study designs, or are you only searching for studies that compare group already? | We are searching for studies that have comparison groups within the study. We will not be comparing groups across different studies. |
| Do you have search terms already prepared, if so what are they? | These are now included in the manuscript in the Strand 1: Scoping Review subsection of the Methods and Analysis section |
| What are the inclusion and exclusion criteria? | These are now included in the manuscript in the Strand 1: Scoping Review subsection of the Methods and Analysis section |
| What data will be extracted to conduct the group comparison? | These are now included in the manuscript in the Strand 1: Scoping Review subsection of the Methods and Analysis section |
| How will the analysis be conducted? | In line with Arksey and O'Malley the data will be extracted and charted.<br><br>Arksey H, O'Malley L. Scoping studies: towards a methodological framework. Int J Soc Res Methodol 2005;8:19–32 |
| There are many other methodological questions will arise as you elaborate on the above questions, more detail on what exactly you intend to do is needed in this study. | Thank you for your suggestion, we have provided detail on the practicalities of conducting the scoping review to address the comments above. The methods section is now more detailed. |

| | |
|---|---|
| Study 2. you indicate that you will use a panel of individuals who have experienced maternal incarceration to identify individuals who have experienced maternal incarceration. It is a good practice to include individuals with lived experience on the panel but it does not get around the issue of how are you going to identify and recruit members to the panel? why are the panel not included in the interviews?<br>How are you going to assess homogeneity and heterogeneity within and between groups and what does it add to separate groups in this way? you mention you will split this across gender but a man who has experience maternal incarceration age 7 possibly will have more in common with a women who also experienced maternal incarceration aged 7 rather than another man who experienced maternal incarceration at 16. How big are these groups?<br>Again going back to the literature review, what gap in the literature do these focus groups address and what is the specific research question of this study? | There was no conscious decision to exclude panel individuals. We are drawing on a pre-existing panel of which not all will have experience of parental imprisonment as children.<br><br>Given this is a small study, we are unable to generalise and we will not be comparing groups. Therefore, we have removed the sentence on homogeneity and heterogeneity instead emphasising the importance of breadth rather than representativeness. We are separating groups not for comparison (outcomes) but to cultivate psychological safety in a small setting<br><br>The gap these focus groups address is the absence of knowledge on the experiences of children with parental imprisonment in terms of how their health needs are addressed and ways that this could be improved. The literature reveals what researchers think is important (or possible) to measure. The qualitative work aims to explore what people with lived experience think is important to measure. |
| Study 3 looks at the experiences of children whose mother is resident in Hope Street. The main area of clarification is what is being addressed here and how does it relate to the previous two studies? The stated aim of Hope street is to reduce the number of children taken into care, what is the aim of this study? is it to demonstrate children are better off or that children are not being taken into care? More work is need to explain how all of these studies are linked together or they need to be submitted as separate protocols with separate aims and objectives. | Thank you for your suggestion of clarity. The aim of this strand (sub-unit) of the study is to inform the development of outcome indicators through generating children-centred data. Interviewing these children enables an exploration of what they consider important for their wellbeing. |
| Study 4, looks at professional perspectives, similar questions are present to study 3 above. Additionally, as you are recruiting professional stakeholders, is this a sort of process evaluation? are you recruiting professionals who have experience of other contexts? what is the over all aim of this arm | As above, the strands are staged building towards the development of outcome indicators rather than separate studies. This is not an evaluation; the professionals are being interviewed not to assess a mechanism of change but to bring expertise on identifying the health needs of the children and how best to measure them. |

| | |
|---|---|
| of the protocol and how does it link back to previous studies? | |
| Study 5. This is an interesting task, why is it being done at the end rather than the beginning? If you identify outcomes to be measured now how are you going to measure them if you have already collected the data? | This is due to the staged approach i.e. multiple components of qualitative work together with the scoping review evidence feeding into the development of indicators. The literature review and the qualitative work will provide a great deal of information on what outcomes researchers have measured, and what outcomes those with lived experience and the professionals working with them feel should be measured. Therefore it is important this final strand is last as the previous work informs it.<br><br>We will not be applying the measures in this study. The purpose of the study is to develop the measures. |
| **Reviewer 2 comments** | |
| This is very important work, and an intriguing study design; however, the protocol leaves gaps in my understanding of how the proposed projects will address the gaps in the literature around children of imprisoned mothers and the overall design is unclear. While the topic itself does seem important, the introduction does not clearly set up how the study will address gaps in our understanding; it is unclear how the different "strands" of the protocol will be combined; and justification for the planned approach is not adequately provided. Below, I have made recommendations to help strengthen the manuscript, grouped into major and minor revisions | Thank you for your careful consideration and highlighting areas for development.<br><br>We agree and have both made the link between the gap in literature clearer and how the study will address these gaps now detailed in the Introduction section.<br><br>The strands are now explicitly described in terms of how they work together with the planned approach reflected in additional content presented in the Methods and Analysis section. |
| Major:<br>- The introduction is compelling regarding the potential harms of having an imprisoned mother, and the large gaps in understanding of children's needs; however, it does not clearly set up why the study proposal focuses on adult children of imprisoned mothers and current children of mothers at Hope Street – which appears to be an incarceration alternative. Please re-format the introduction to set up clear gaps in the literature that are then directly addressed by what is proposed in this study protocol.<br><br>- The scoping review seems to be more of a background literature search to inform the | Thank you for highlighting the points. There is a lack of understanding on the experiences of children with maternal imprisonment and separation through justice involvement in terms of how their health needs are addressed and ways that this could be improved. The children of mothers at Hope Street have experience of separation through justice-involvement (alternative of Hope Street), some may also have maternal imprisonment also (prior to Hope Street).<br><br>The introduction section now states the gaps and how they are directly addressed by what is proposed by the study protocol. |

| | |
|---|---|
| main study; it is not clear to me it should be described here.<br>- Further, the scoping review protocol is limited – what data will be collected? How will this inform strands 2 and 3<br>- Please provide justification for the study design, particularly strands 2 and 3. For example, why 2-3 focus groups? How will you decide? What is the goal of the workshop? How many people will be there?<br>- The descriptions of all strands lack detail, please add.<br>- What is the goal of the in-depth interviews? What data are you hoping to obtain from 8+ year olds?<br>- What is the goal of focus groups and workshops? | All six points here on methods have been addressed and this detail is in the expanded Methods and Analysis section. |
| Minor:<br>- Abstract re: 17,000 children, please contextualize – is this in the UK broadly or a particular area?<br>- Intro, first paragraph, is there evidence to support that children are likely to move area and start a new school if their mother is imprisoned? If so, cite.<br>- The use of "etc." throughout the document feels inappropriate.<br>- Dates of the study missing | Thank you for these smaller suggestions. Each has been addressed and in order can be found in the abstract, introduction sections. 'etc.' has been removed throughout and the date has been inserted into the start of the Methods and Analysis section. |

## VERSION 2 - REVIEW

| | |
|---|---|
| **Reviewer** | **2** |
| **Name** | **Holaday, Louisa** |
| **Affiliation** | **Mount Sinai School of Medicine** |
| **Date** | **05-Jan-2026** |
| **COI** | |

I again commend the authors for this important work, and appreciate their efforts to address prior reviews. However, as a reader I still find the protocol a bit disjointed and lacking in details. Below some suggestions to strengthen:

Major /

- Intro as currently written seems to include findings from scoping review, eg difficult to compare across studies

- The intro still does not clearly set up the study questions, at least for me. I think this is because the authors are studying both current and former children of incarcerated mothers but scoping the literature on the differences between fathers and mothers rather than just what is known about mothers. Further, the authors are doing a lot of work to assess the alternatives to incarceration program, Hope street, but do not adequately justify this approach in understanding the health effects of maternal imprisonment.

- Still lacking in justification for the number of focus groups

- I don't understand how focus groups with those with formerly imprisoned mothers will inform interviews of children whose mothers are at the alternative to incarceration site where the children will be able to be with them?

- It remains unclear what information the authors are hoping to obtain from 8yo children in these interviews. What is the justification for speaking with children this young?

- There is still inadequate justification for the methods (some specific points below)

Minor / abstract: what is an outcomes workshop? What is PPIE?

- intro is missing refs on mother likely to have been primary carer and 95% remain with mother if father is imprisoned

- is there a ref that they are likely to move area? Or can that comment be softened "may move area"?

- also need a reference regrading permanent loss of home

- scoping review – were only studies looking at people with both mother and father incarcerated included? Intervention inclusion and exclusion criteria seem to contradict each other

- scoping review – description says not including unpublished, but table does

- how are the number of interviewees determined? How many from Hope street and how many nationally with imprisoned mothers?

- Need more detail on how the workshop data will be analyzed

- Why were qualitative studies not included in the scoping review?

---

## VERSION 2 - AUTHOR RESPONSE

**Requested revisions** **Responses to reviewer suggestion**

| **Reviewer 1** | |
|---|---|
| Intro as currently written seems to include findings from scoping review, eg difficult to compare across studies | Thank you for this comment. It is a fair point given the inevitable background literature overlap with the literature used for the Scoping Review (ScR). We have removed the granular content which features as findings in the ScR |

| | |
|---|---|
| | which hopefully gives a clearer read (p.5-6). Please note: the page numbers refer to the marked copy submitted |
| The intro still does not clearly set up the study questions, at least for me. I think this is because the authors are studying both current and former children of incarcerated mothers but scoping the literature on the differences between fathers and mothers rather than just what is known about mothers. Further, the authors are doing a lot of work to assess the alternatives to incarceration program, Hope street, but do not adequately justify this approach in understanding the health effects of maternal imprisonment | Thank you for your careful consideration. We have reworked the section, adding in prose (p.7) so that the content better signals to each of the study questions (objectives), understanding gendered differential impacts of parental imprisonment , ii) qualitative data with children with lived experience to shape the indicator development iii) and understand the potential service provision needs<br><br>As previous scoping work had focused on parental imprisonment, we build on this by teasing out the specificities between mothers and fathers to deepen understanding relating to mothers. I have reflected this in the manuscript p.5<br><br>The qualitative work is to understand the health impacts of those with experience of maternal separation through maternal justice involvement/imprisonment. Hope Street provides an opportunity to directly engage with this this invisibilised population as many of the referred women have histories of imprisonment. This is reflected on p. 9 |
| Still lacking in justification for the number of focus groups | Thank you, this justification has now been added (p.8) |
| I don't understand how focus groups with those with formerly imprisoned mothers will inform interviews of children whose mothers are at the alternative to incarceration site where the children will be able to be with them? | The themes emerging from the focus groups will inform the topic themes for the interviews with the young children due to the shared experience of maternal imprisonment and probation across these two groups. I have reflected this in the manuscript, on p.8 |
| It remains unclear what information the authors are hoping to obtain from 8yo children in these interviews. What is the justification for speaking with children this young? | This is such an important point. These children are directly impacted and therefore the value of speaking to them is immense insight given their lived realities of health and wellbeing and maternal imprisonment. I have cited this with supporting literature on the manuscript (p.5) |
| Minor / abstract: what is an outcomes workshop? What is PPIE? | I have updated 'outcomes workshop' to become 'outcomes development workshop' for clarity and added the full phrase of public and patient involvement and engagement. Patient and Public Involvement and Engagement (PPIE) is a well established approach in UK health research which funders and service providers have embedded in their work in recent decades to centre the |

| | |
|---|---|
| | expertise of lay people in decision-making. Supporting literature has been added |
| Intro is missing refs on mother likely to have been primary carer and 95% remain with mother if father is imprisoned | This has been rectified, thank you |
| Is there a ref that they are likely to move area? Or can that comment be softened "may move area"? | Thank you for this suggestion, actioned |
| - also need a reference regarding permanent loss of home | Reference has been added here, thank you |
| scoping review – were only studies looking at people with both mother and father incarcerated included? Intervention inclusion and exclusion criteria seem to contradict each other | We included studies that had outcomes relating to health and behavioural health of an individual who had experienced maternal or paternal imprisonment. I have reflected this on the manuscript for clarity (p.6) |
| how are the number of interviewees determined? How many from Hope street and how many nationally with imprisoned mothers? | Thank you, this is now detailed |
| scoping review Description says not including unpublished, but table does | Thank you for flagging this inconsistency which Is now rectified |
| - Need more detail on how the workshop data will be analyzed | Analysis details has now been added |
| **Reviewer 2** | |
| If you have selected 'Yes' above, please provide details of any competing interests.: not applicable | Unclear on the suggested amendment here. I have double checked the competing interests section. |

## VERSION 3 - REVIEW

| | |
|---|---|
| **Reviewer** | **2** |
| **Name** | **Holaday, Louisa** |
| **Affiliation** | **Mount Sinai School of Medicine** |

**Date** **24-Mar-2026**

**COI**

---

Overall, kudos to the authors for a great revision – things are much clearer now. A few mostly minor things:

Major /

- To me, it still is unclear how a scoping review comparing maternal and paternal imprisonment sets up the rest of your study better than just a scoping review of health effects of maternal imprisonment on children. It seems the specificity of your review will limit the knowledge you gain about maternal incarceration to inform your further work. But this may be my lack. You explain what you are doing clearly so I don't think further revision is necessary.

Minor /

- Are qualitative studies excluded from the scoping review? If so, please clarify why

- For the interviews, it is still unclear why you will include children from Hope Street whose mother has never been imprisoned. You say many may have been in the past, but why are you including children who have never experienced maternal imprisonment?

- Consider in abstract something about # with mother otherwise involved in CJS

- Are ACEs known to have an "impact" rather than just an "association" with long term health and well-being?

---

## VERSION 3 - AUTHOR RESPONSE

CHAMPION study protocol: children whose mothers are involved in the criminal justice system in Dorset & Hampshire, United Kingdom: developing health and social care outcome indicators

The authors would like to thank the reviewers for their constructive comments. Please see our responses to the minor revision requests below:

Major /

- To me, it still is unclear how a scoping review comparing maternal and paternal imprisonment sets up the rest of your study better than just a scoping review of health effects of maternal imprisonment on children. It seems the specificity of your review will limit the knowledge you gain about maternal incarceration to inform your further work. But

this may be my lack. You explain what you are doing clearly so I don't think further revision is necessary.

- Thank you for this observation, we take your point. By extending our focus to the differential effects we were arguably opening up the potential to bring greater insights in relation to maternal imprisonment effects than exclusively focusing on maternal imprisonment.

Minor /

1) Are qualitative studies excluded from the scoping review? If so, please clarify why.

To ensure robust mapping of measurable population-level outcomes and associations qualitative studies were excluded from scoping. The inclusion criteria of prevalence studies, cross-sectional studies, cohort studies, case control studies, surveys was most appropriate to enable a comparable epidemiological evidence base. This clarification has been added into the manuscript.

2) For the interviews, it is still unclear why you will include children from Hope Street whose mother has never been imprisoned. You say many may have been in the past, but why are you including children who have never experienced maternal imprisonment?

The Hope Street mothers faced criminal justice sentencing, some being imprisoned and others being served suspended/community order sentences. Whilst the children may not have experienced their mother being imprisoned, they will have experienced their mother's arrest and subsequent court proceedings and other processes of community punishment, such as probation supervision and other court order requirements. This clarification has been added into the manuscript.

3) Consider in abstract something about # with mother otherwise involved in CJS

Thank you for this suggestion, this is one of the major routine data gaps. There are no official or NGO statistics for this invisibilised population. We have added a sentence in making this clearer in abstract,

4) Are ACEs known to have an "impact" rather than just an "association" with long term health and well-being?

Thank you for flagging this, the language has been changed in the manuscript to association.

Reviewer: 2

If you have selected ‘Yes’ above, please provide details of any competing interests.: not applicable. Checked.

We hope these now meet the requirements for publication and again thanks to the reviewers and editors for supporting the development of the manuscript.
